# Effects of Creatine Supplementation on Lower-Limb Muscle Endurance Following an Acute Bout of Aerobic Exercise in Young Men

**DOI:** 10.3390/sports8020012

**Published:** 2020-01-21

**Authors:** Itamar P. Vieira, Amanda G. de Paula, Paulo Gentil, Claude Pichard, Darren G. Candow, Gustavo D. Pimentel

**Affiliations:** 1 Clinical and Sports Nutrition Research Laboratory (Labince), Faculty of Nutrition, Federal University of Goias, Goiânia 74605-080, Brazil; itamarpef@gmail.com (I.P.V.); mnd.gms1@gmail.com (A.G.d.P.); 2 Faculty of Physical Education and Dance, Federal University of Goias, Goiânia 74605-080, Brazil; paulogentil@hotmail.com; 3 Clinical Nutrition, Geneva University Hospital, 1205 Geneva, Switzerland; claude.pichard@unige.ch; 4 Faculty of Kinesiology and Health Studies, University of Regina, Regina, SK S4S OA2, Canada; darren.candow@uregina.ca

**Keywords:** creatine, aerobic exercise, concurrent exercise, strength loss, muscle

## Abstract

We aimed to determine whether creatine supplementation influences lower-limb muscle endurance following an acute bout of aerobic exercise (AE) in young healthy men. Using a randomized, double-blind, placebo-controlled crossover design, 11 men (26.5 ± 6.2 years, body mass index 26.6 ± 2.1 kg/m^2^),with 12 months of experience in strength training (three times/week) and AE (two times/week) were randomized to receive creatine (20 g/day plus 20 g/day maltodextrin) and placebo (40 g/day maltodextrin) for 7 days, separated by a washout period of 14 days, before performing an acute bout of AE (30 min on treadmill at 80% baseline maximum velocity) which was followed by four sets of bilateral leg extension endurance exercise using a 10-repetition maximum protocol (10 RM)). There was a significant decrease in the number of repetitions performed in the third (Placebo: −20% vs. Creatine: −22%) and fourth set (Placebo: −22% vs. Creatine: −28%) compared with the first set (*p* < 0.05), with no differences between creatine and placebo. Additionally, no differences were observed between creatine and placebo for the total number of repetitions performed across all four sets (Placebo: 33.9 ± 7.0 vs. Creatine: 34.0 ± 6.9 repetitions, *p* = 0.97), nor for total work volume (Placebo: 3030.5 ± 1068.2 vs. Creatine: 3039.8 ± 1087.7 kg, *p* = 0.98). Short-term creatine supplementation has no effect on lower-limb muscle endurance following an acute bout of aerobic exercise in trained young men.

## 1. Introduction

Concurrent exercise (CE) involves the combination of aerobic exercise (AE) and resistance training in the same training session [1]. CE is common practice among exercising individuals and athletes and increases physical performance and body composition [2,3,4,5,6]. However, previous studies have shown that performing AE before resistance training results in acute decreases in muscle performance [2,4,7,8,9,10], possibly due to an increase in peripheral fatigue, AMP-activated protein kinase (AMPK) and Peroxisome proliferator-activated receptor gamma coactivator 1-alpha (PGC-1α) signaling and decrease in satellite cell activity [1].

Supplementation with creatine, an organic acid derived from reactions involving arginine, methionine, and glycine in the kidney and liver [1], has been shown to increase muscle performance, possibly by influencing high-energy phosphate metabolism, satellite cell activity, muscle protein kinetics, and inflammation [11,12]. Theoretically, creatine supplementation may be an effective nutritional intervention following AE to maintain muscle performance. For example, Painelli et al. [4] showed that creatine supplementation (20 g/day for 7 days + 5 g/day thereafter) maintained lower-body muscle endurance (number of repetitions performed) following acute bouts of intermittent and continuous AE in strength-trained males compared with a decrease in males who received placebo. However, this study was limited by the parallel group design.

The purpose of this study was to determine whether creatine supplementation maintains muscle performance following an acute bout of AE in trained young males using a randomized, cross-over design. Cross-over designs typically reduce the influence of confounding variables on the dependent outcome measures and are considered more statistically powerful (less variance) compared with parallel group designs. Based on the mechanistic actions of creatine and the findings of Painelli et al. [4], it was hypothesized that creatine supplementation would maintain lower-limb muscle endurance following an acute bout of AE compared with placebo in trained young males.

## 2. Methods

### 2.1. Participants

Seventeen men with 12 months of strength training (three times per week) and AE (two times per week) experience volunteered. Participants were excluded if they were vegetarian, had consumed protein or creatine supplements six months prior to the start of the study, if they had a history of hormonal therapy interventions or anabolic steroid use, or if they had pre-existing kidney or liver abnormalities. Participants were instructed not to change their diet or physical activity patterns during the study. Participants were informed of the risks and discomforts associated with the study before providing written consent. Experimental design was approved by the Research Ethics Committee (no. 2.507.216), and after establishing the inclusion and exclusion criteria, the participants signed the inform consent form.

### 2.2. Study Overview

**Figure 1:**
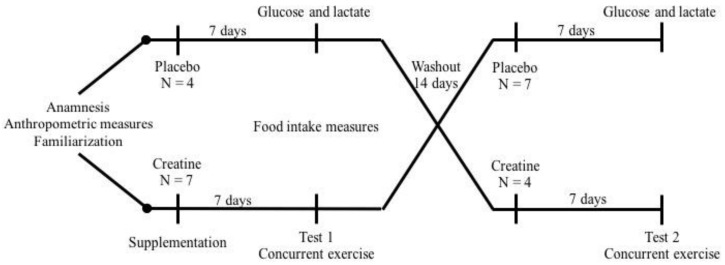
Experimental design. After anamnesis, anthropometric evaluation, strength tests, and exercise familiarization, eleven participants on a high-protein diet and placebo or creatine supplementation for one week were submitted to acute concurrent exercise session. After a washout period of fourteen days, the same protocol was repeated.

The study was a double-blind, placebo-controlled crossover trial where participants were randomized using a computer-generated schedule (https://www.randomizer.org/) to consume creatine and placebo for 7 days, separated by a 14 day washout period. After each 7 days supplementation phase, participants performed an acute bout of AE experimental test consisting of a 30 min run on a treadmill (Technogym^®^, Excite Run 1000, Cesena, Italy) at 80% maximum velocity (MV) obtained in the test. Immediately following the treadmill exercise, participants performed four sets of bilateral leg extension exercise (Technogym^®^, Leg Extension Med, São Paulo, Brazil) with the load obtained on the 10-repetition maximum (10 RM) test. All sets were performed to momentary muscle failure as previously defined [13]. Rest between sets was 2 min. Blood glucose and lactate concentrations were determined before and after the acute bout of AE tests. During the 14 days washout period, no supplement was consumed (Figure 1). Prior to the acute bout of AE, participants were instructed to abstain from alcohol, caffeine, other supplements, and strenuous exercise for 48 h. Participants arrived for testing 1 h after their last meal and pre-test feeding was standardized (yogurt with banana). Ad libitum water consumption was allowed during the tests and food intake was measured using three 24 h food recalls. Prior to randomization and supplementation, participants performed a familiarization trial with the exercise equipment to reduce the amount of learning which may have contributed to our findings.

### 2.3. Supplementation

Participants ingested 20 g of creatine monohydrate (20 g; Max Titanium^®^, Supley, Matão, Brazil; 99.9% purity) and 20 g maltodextrin or 40 g of maltodextrin (placebo, Max Titanium^®^, Supley, Matão, Brazil) for 7 days. After the 14 days washout period, participants crossed-over and consumed the opposite supplement for 7 days. The total daily amount of supplement was divided into four equal portions and consumed with food throughout the day. Creatine and placebo were identical in taste, color, texture, and appearance. Supplement packages were unmarked so neither the participant nor the researcher knew the content.

### 2.4. Anthropometric Measures

Body mass was measured using a digital personal scale (HN-289LA^®^ Omron Healthcare Co., Muko, Kyoto, Japan) and height using a portable stadiometer (Sanny^®^, São Paulo, Brazil), and body mass index (BMI) was then calculated. Upper- and lower-limb and waist circumference was measured twice using a tape measure. Skinfold thicknesses (subcutaneous adipose tissue) were measured using a caliper (Lange^®^ Skinfolder Caliper, Beta Technology, Santa Cruz, USA) and body fat was calculated according to the Jackson and Pollock protocol [14]. Anthropometric assessments were performed by the same trained researcher.

### 2.5. Dietary Intake Analyses

Dietary intake was assessed by having participants fill out three 24 h food diaries on separate days (two weekdays and one weekend day) to evaluate habitual food consumption [15]. The dietary intake analysis consisted of total calories, carbohydrate, lipids, proteins, leucine, valine, and isoleucine. Food intake calculus was performed using the DietPro^®^ software (version 6.0, Viçosa, Brazil) using the Food Database Table of the United States Department of Agriculture [16].

### 2.6. Maximum Graded Test

A maximal graded exercise test was performed on a treadmill (Technogym^®^, Excite Run 1000, Cesena, Italy), with slope set at 1%. After a warm-up that consisted of walking at 6.0 km/h for 3 min, the treadmill was adjusted with the speed of 8.0 km/h, followed by an increase of 1.0 km/h in each subsequent minute until the participants reached exhaustion. The velocity at the last complete stage before exhaustion was record as the MV. Participants were strongly encouraged verbally to exert maximum effort [17].

### 2.7. Maximum-Repetition Strength (10 RM) and Strength Endurance Test

The 10-repetition maximum (10 RM) test was performed using the leg extension machine (Technogym^®^, Leg Extension Med, São Paulo, Brazil). The procedures followed the recommendations previously described [10,18]. The participants performed the warm-up with ten repetitions performed at a self-selected comfortable load. After a rest of 5 min, the estimated 10 RM load was adjusted based on the training history of each participant. If the volunteer was not able to perform ten repetitions or performed more than ten repetitions, the load was adjusted for the next attempt. Only three attempts were allowed, with rest of 5 min between them. The 10 RM loads were obtained for all participants in two to three attempts. Participants performed the tests with their backs in contact with the support and were not allowed to use trunk movements or raise their hips from the chair. The tests were stopped when the participants were unable to do the movement properly (total range of motion without changes in the technique) for two consecutive repetitions. The familiarization of strength endurance test involved of the conclusion of four sets to failure at 80% of the load as per the protocol published previously [4]. The tests were performed by trained professionals and verbal motivation was used in all sets.

### 2.8. Biochemical Analysis

Blood lactate was measured using a portable lactometer (Accutrend^®^ Plus; Roche Accutrend Plus, New York, NY, USA). Blood glucose was measured by digital glucose meter (Accu-chek^®^ Active; Roche, São Paulo, Brazil). All blood samples were taken from the finger by a trained professional.

### 2.9. Statistical Analyses

The normality of the data was tested using the Kolmogorov–Smirnov test. General characteristics, dietary food intake, leg extension repetitions, and blood lactate concentrations are presented as mean ± standard deviation and glucose levels are presented as median (minimum and maximum). Strength data were analyzed using the two-way ANOVA followed by Tukey test. The unpaired *t* test was used to compare the total work volume and blood lactate concentrations between groups. The Mann–Whitney test was used to compare the delta blood glucose concentrations. The Fisher exact test was performed to assess the rate of participants who correctly guessed their allocation in the group. All statistical analyses were done using the MedCalc^®^ Seoul, Korea, software, and *p* < *0*.05 was defined as significant difference.

## 3. Results

Of the 17 participants who initially volunteered, six were excluded for not adhering to the proper supplementation protocol. Therefore, results from 11 participants were used in the analyses. (Table 1). Prior to starting the study, all participants from both groups ingested a low-carb (3.0 ± 1.0 g/kg/day) and high-protein diet (1.5 ± 0.3 g/kg/day), with no difference in dietary intake between intervention periods (Table 2). No side effects were reported from the supplementation or exercise intervention. Verbal confirmation of supplementation compliance was 100%.

**Figure 2:**
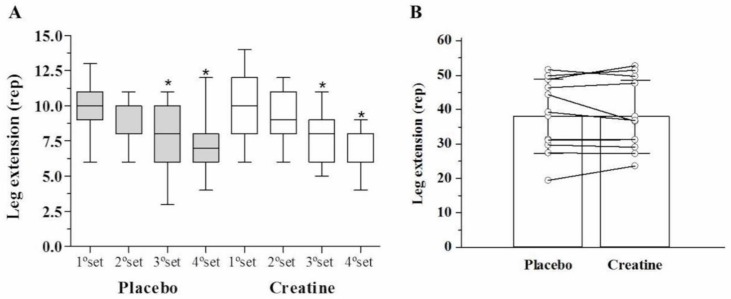
Strength endurance in leg extension (repetitions) among the sets (**A**) and leg extension (sum of repetitions) (**B**).

**Figure 3:**
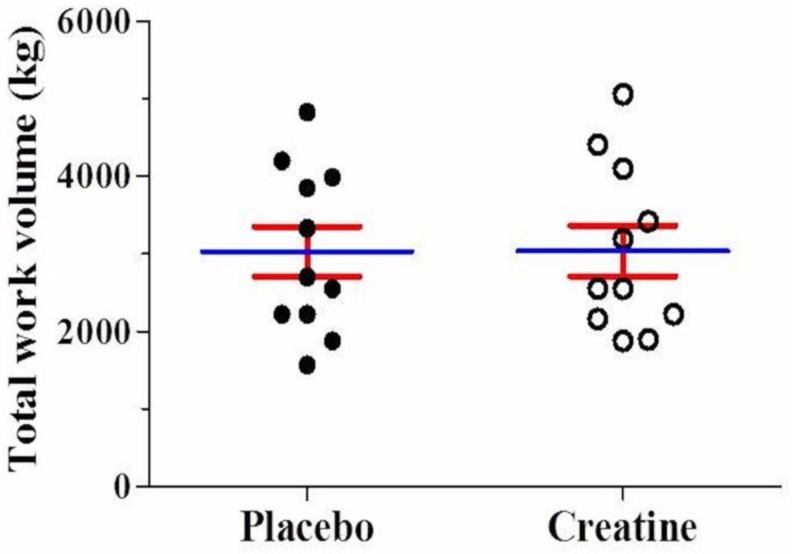
Mean value for total work (kg) using the leg extension machine either on placebo or creatine supplementation.

After AE, there was a significant reduction (*p* < 0.05) in leg extension muscle endurance (number of repetitions performed) in the third (Placebo: −20% vs. Creatine: −22%) and fourth set (Placebo: −22% vs. Creatine: −28%) compared with the first set. However, there were no differences between creatine and placebo (Figure 2A). Across all four sets, no differences were observed in the total number of repetitions performed (Placebo: 33.9 ± 7.0 vs. Creatine: 34.0 ± 6.9 repetitions, *p* = 0.97) (Figure 2B). Additionally, no difference in total work volume was found between creatine and placebo in kg (Placebo: 3030.5 ± 1068.2 vs. Creatine: 3039.8 ± 1087.7 kg, *p* = 0.98) (Figure 3) and joules (Placebo: 3030.4 ± 1068.2 vs. Creatine: 3035.5 ± 1092.8 J/m, *p* = 0.99).

There were no significant differences between creatine and placebo for changes in delta blood glucose (Placebo: 5.0 (−73.0–67.0) vs. Creatine: 1.0 (−53.0–49.0) mg/dL, *p* = 0.73) and blood lactate (Placebo: 5.1 ± 2.9 vs. Creatine: 7.9 ± 4.9 nmol/L, *p* = 0.11) concentrations (see Supplementary Figure S1).

Regarding supplement blinding efficacy, 6/11 participants correctly guessed when they were consuming placebo and 5/11 correctly guessed when they were consuming creatine, which was not statistically different (*p* = 1.00).

## 4. Discussion

There are two hypotheses for the reduction in muscle endurance following AE, (i) acute; peripheral fatigue triggered by muscle damage and glycogen depletion during AE training reduces the ability of skeletal muscle to produce tension during resistance training [2,3], and (ii) chronic; skeletal muscle attempts to adapt to both forms of training, however, morpho-functional adaptations, such as fiber type and size after endurance exercise and weight training are partially opposed resulting in an interference effect.

The current study aimed to assess the influence of creatine supplementation on muscle endurance following an acute bout of AE in trained young males. Results showed that creatine had no effect on muscle endurance or total work performed which is in contrast to the findings of Painelli et al. [4], who showed that creatine supplementation (20 g/day for 7 days + 5 g/day thereafter) maintained lower-body muscle endurance (number of repetitions performed) following acute bouts of intermittent and continuous AE in strength-trained males (n = 15) compared with a decrease in males (n = 16) who received placebo. The authors suggest that the increased availability of phosphoryl creatine and its potential buffering capacity (reduction of H^+^ ions) would be responsible for maintaining muscle endurance in the legs. Furthermore, in females who performed a leg-press 1 RM prior to and immediately following an acute bout of endurance exercise, there was a positive effect from creatine supplementation on the performance of four sets of leg press at 80% of 1 RM [8]. While it is difficult to compare results across studies, methodological differences may be involved. In the Painelli et al. [4] study, leg press and chest press muscle endurance (both multi-joint exercises) was assessed whereas we only assessed leg extension endurance (single-joint exercise). Furthermore, females were assessed in the study by Aoki whereas we assessed only males. Previous studies have shown differences in muscle fatigability [6,8,19] and responsiveness to creatine supplementation between sexes [20,21].

Additionally, no difference in blood lactate concentrations was reported. These data are similar to those from a previous study [22].

Our data show no positive effect of creatine supplementation on muscle strength using a crossover design and with dietary control during the study. Considering that the participants were on a high protein-diet, the creatine supplementation might be not necessary. This might be explained because protein ingestion can help in muscle recovery [23] and might influence recovery from aerobic activities. However, creatine supplementation it seems did not bring additional benefits in men who intake a high-protein and low-carb diet.

A recent study [5] investigated a chronic protein supplementation effect (6 months) on muscle strength in sedentary women and men on CE. Men who ingested protein supplementation 2.2 g/kg/day, showed higher increases in strength in the bench press when compared with the group that ingested 1.1 g/kg/day of protein. It is interesting to note that men who received a high-protein (2.2 g/kg/day) group ingested lower carbohydrate (not low-carb diet) than the normal protein (1.1 g/kg/day) group. On the other hand, in well-trained male cyclists who performed an acute exercise session (high-intensity cycling and 100 drop-jumps), 20 g hydrolysate protein supplementation associated with a habitual high-protein diet (1.2 g/kg/day) and moderate in carbohydrate (6 g/kg/day) did not alleviate exercise-induced muscle damage [7].

Although training enhances the effectiveness of supplementation protein during resistance exercise [24], the effects of habitual consumption of a high-protein diet on muscle strength during concurrent training are limited [5]. It would be interesting if future studies evaluate the effects of creatine supplementation under high- and low-habitual protein and carbohydrate intakes.

Although we did not measure the timing of protein intake, no significant effect on muscle strength is found during the resistance training [25]. Thus, further studies are warranted to examine the effects of a high-protein diet on muscle strength during a CE bout.

### Study Limitations

There were several limitations to this study. First, we used a 14 days washout period between creatine and placebo ingestion which may not have been long enough to abolish the residual (carry-over) effects of creatine. For example, Vandenberghe et al. [26] showed that creatine supplementation (20 g/day for 4 days) increased intramuscular PCr concentrations, which were maintained with a maintenance dosage of creatine (5 g/day) for 10 weeks. Upon creatine cessation, intramuscular PCr concentrations remained elevated for 28 days. Second, no measure of intramuscular creatine (PCr, free Cr) was assessed prior to each testing phase. Initial intramuscular creatine levels typically determine the responsiveness to creatine supplementation [27]. Third, participants may have already been consuming high amounts of dietary creatine from protein-containing food products (i.e., seafood, meat, poultry) [28,29], which attenuated the ergogenic response to creatine supplementation. Food records prior to the start of supplementation showed that participants were consuming approximately 1.5 ± 0.3 g/day of protein. Unfortunately, the food diaries did not determine the amount of dietary creatine consumed. Fourth, the majority of intramuscular creatine is found in type II muscle fibers. Young individuals with the highest concentration and muscle cross-sectional area of type II fibers respond more favorably to creatine supplementation [30]. Unfortunately, no measure of muscle fiber morphology was made in this study. Finally, the absence of positive effect found could be due to the fact of a small sample size. Thus, a study with a large sample size and participants with different training stages should be explored in the future.

## 5. Conclusions

In summary, short-term creatine supplementation has no effect on lower-limb muscle endurance following an acute bout of AE in trained young males. Our results show new information regarding muscle strength recovery after an acute bout of AE and raise a hypothesis that the increase in carbohydrate intake combined with the high-protein diet should be investigated.

## Figures and Tables

**Table 1:** Participants’ characteristics.

Characteristics	Mean ± SD
Age (years)	26.5 ± 6.2
Body weight (kg)	77.6 ± 7.2
Height (m)	1.7 ± 0.0
Body mass index (kg/m^2^)	26.6 ± 2.1
Body fat (%)	14.4 ± 6.6
Work volume leg extension (kg)	88.1 ± 18.6
Total work volume (kg)	3030.5 ± 1068.2
Effort time run for 5 km (min)	25.5 ± 2.6

**Table 2:** Dietary food intake.

Nutrients	Mean ± SD
Total calories (kcal)	2196.6 ± 702.9
Carbohydrate (%)	43.6 ± 8.2
Carbohydrate (g/kg)	3.0 ± 1.0
Protein (%)	26.4 ± 4.3
Protein (g/kg)	1.5 ± 0.3
BCAA (g)	19.0 ± 6.1
Leucine (g)	8.3 ± 2.9
Valine (g)	5.8 ± 1.8
Isoleucine (g)	4.8 ± 1.5
Lipids (%)	29.9 ± 9.5

BCAA: Branched-chain amino acids.

## Supplementary Materials

The following are available online at https://www.mdpi.com/2075-4663/8/2/12/s1, Figure S1: Delta blood glucose (**A**) and lactate (**B**) concentrations for participants either on placebo or on creatine supplementation. No significant differences observed between groups.
