# Supplementary material for: Effects of Creatine Supplementation on Lower-Limb Muscle Endurance Following an Acute Bout of Aerobic Exercise in Young Men
Source: Sports (Basel). 2020 Jan 21;8(2):12. doi: 10.3390/sports8020012 (PMC7077271; doi:10.3390/sports8020012)
Supplement: Supplementary file 1 [file sports-08-00012-s001.pdf]

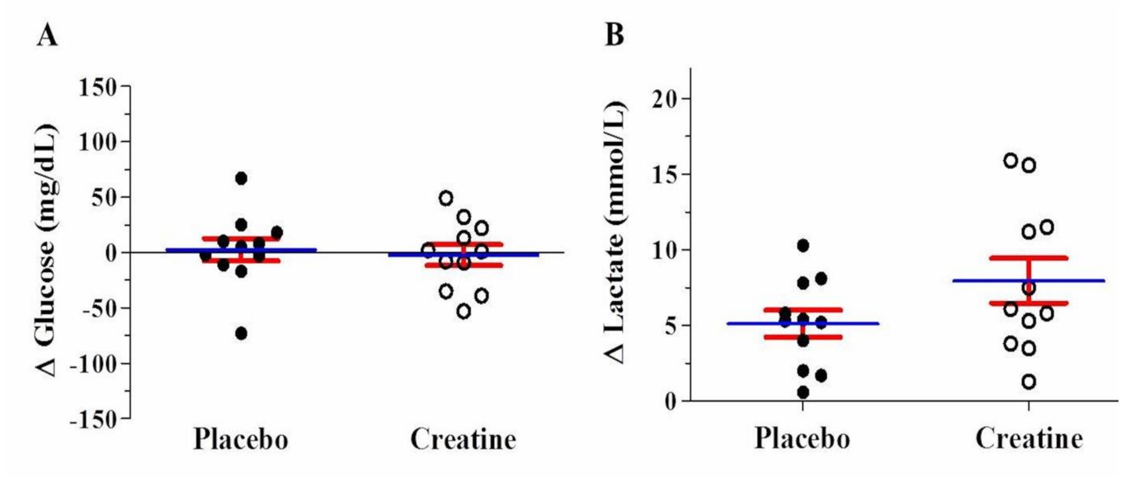

Figure S1. Delta blood glucose (**A**) and lactate (**B**) concentrations for participants either on placebo or on creatine supplementation. No significant differences observed between groups.
